# Bioecological Parameters of the Black Fig Fly, *Silba adipata* (Diptera: Lonchaeidae), Collected from Fig Crops in Mexico

**DOI:** 10.3390/insects15110883

**Published:** 2024-11-11

**Authors:** Eduardo Paniagua-Jasso, Manuel Alejandro Tejeda-Reyes, Ana Mabel Martínez-Castillo, José Isaac Figueroa-de la Rosa, Diana Vely García-Banderas, Luis Jesús Palma-Castillo, Carlos Patricio Illescas-Riquelme, Samuel Pineda-Guillermo

**Affiliations:** 1 Instituto de Investigaciones Agropecuarias y Forestales, Universidad Michoacana de San Nicolás de Hidalgo, Carretera Morelia-Zinapécuaro, Km. 9.5, Tarímbaro 58880, Michoacán, Mexico; 0856956h@umich.mx (E.P.-J.); ana.martinez@umich.mx (A.M.M.-C.); jose.figueroa@umich.mx (J.I.F.-d.l.R.); ljpalmac@gmail.com (L.J.P.-C.); 2 Programa de Postgrado en Protección Vegetal, Universidad Autónoma Chapingo, Carretera México-Texcoco, Km 38.5, Chapingo, Texcoco 56230, Estado de México, Mexico; manuel.tejeda.r@gmail.com; 3 Comité Estatal de Sanidad Vegetal de Michoacán, Uruapan 60000, Michoacán, Mexico; dianav.garcia@cesavemich.org.mx; 4 CONAHCYT/Centro de Investigación en Química Aplicada, Departamento de Biociencias y Agrotecnología, Saltillo 25294, Coahuila, Mexico

**Keywords:** invasive pest, *Ficus carica*, cultivar Black Mission, survival and longevity

## Abstract

This study assessed several bioecological aspects of the black fig fly, *Silba adipata* McAlpine (Diptera: Lonchaeidae), the main pest of fig (*Ficus carica* L.). Figs were collected from eight sites in the Mexican states of Michoacán (Plan de Ayala, Los Tejones, Antúnez, Tangancícuaro, Indaparapeo, El Carrizal, and Charapendo) and Morelos (Telixtac). No infestation was recorded in figs collected in Charapendo, but, in the remaining sites, it was variable (2–33%). Figs from Plan de Ayala and El Carrizal were longer and contained more larvae than those from Telixtac and Los Tejones. Some figs (≤12) collected in Telixtac and Los Tejones contained few pupae or adults. The survival of larval and pupal stages (≤86%) and the proportion of females (40–53%) were determined at the sites where the infestation of figs was >6% (Telixtac, El Carrizal, Plan de Ayala, and Los Tejones). In the second part of this study, the development of individuals collected in Los Tejones was analyzed under constant conditions. The estimated larval duration time was between 13 and 15 d, whereas pupae lasted 11 d. The survival rate and longevity of females and males were very similar. Our results could help design a program for integrated pest management against *S. adipata*.

## Simple Summary

The black fig fly *Silba adipata* is an invasive monophagous fly that causes extensive damage to figs where it is cultivated. In addition, this pest limits the marketing of figs due to quarantine restrictions. To manage *S. adipata*, growers constantly apply broad-spectrum insecticides and collect dropped figs; however, these methods of control are not successful. To gain a better understanding of *S. adipata*, in this study, we determined several of their bioecological aspects through a survey taken in eight Mexican sites of commercial fig plantations. There were differences in percentages of infestation, from 0 to 33%, in the different collection sites. Longer figs can host a greater number of larvae. In two sites, 1.1 and 18.2% of figs were found with pupae and adults inside. Larval and pupal survival was very high (≥90 and ≥86%, respectively). The estimated duration of the larval stage was between 13 and 15 d, while the average pupal stage was 11 d. There were no differences in sex ratio or longevity. Survival was 100% up to 12 d. The information generated in this study can be used as a basis for developing integrated management strategies for *S. adipata*.

## 1. Introduction

The fig (*Ficus carica* L.) has its center of origin, diversity, and domestication in the Middle East [1]. However, due to its adaptability, it is cultivated in the subtropical, tropical, and temperate regions of the world [1,2]. The global production of fig is approximately 1,361,981 tons on 302,116 ha of cultivated land, with Turkey, Egypt, Morocco, and Algeria responsible for 65% of the total world production [3]. Figs are consumed both fresh and dried but are also processed into bread, jams, jelly, candy, fig coffee, and fillings for sweet treats [4,5].

The black fig fly, *Silba adipata* McAlpine (Diptera: Lonchaeidae), a severe invasive pest, is considered one of the most important economic threats to figs worldwide [6,7,8,9]. This insect is native to the Mediterranean and Middle East [6], and since 2000, it has spread rapidly throughout temperate areas and has successfully established in Europe, Africa, Asia, and North America [9,10,11,12]. In Mexico, *S. adipata* was detected for the first time in commercial fig orchards in 2019, in the municipality of Ayala, in the state of Morelos, but today it is present in the main fig-producing regions as well [7,13,14,15,16]. In Mexico, 2168 ha of land used for fig cultivation produces 12,489 tons per year; the state of Morelos produces 30% of the total national production [17].

*Silba adipata* is a monophagous species that attacks wild and cultivated figs [2,6]. This insect is multivoltine, producing between four and six generations per year [18]. Females oviposit exclusively beneath the scales, protecting the ostiole of the syconium (i.e., the fig), preferably on unripe figs [18]. After emergence, the larvae enter the fig and feed on the receptacle tissue, just beneath the fig’s skin [8,10]. *Silba adipata* larvae feeding on the figs results in premature fig drop, which is frequently confused by the growers as natural fig shedding [6,9]. Larvae of this insect also feed on ripe figs, causing rot [2] and their rejection for marketing. *Silba adipata* pupates in the soil [8], where it can overwinter [19].

To combat *S. adipata*, growers often use the broad-spectrum insecticides spinosad, bifenthrin, and diazinon, singly or in mixtures weekly [20,21]. However, this method of control has been unsatisfactory, and, in some cases, growers are forced to abandon their fig crops even before the harvest season [22]. In the Mediterranean basin, it has been recognized that applications of chemical compounds cannot be performed in fig orchards because this can result in the elimination of its pollinator wasp, *Blastophaga psenes* L. (Hymenoptera: Agaonidae) [6]. A better understanding of the basic bioecological characteristics of *S. adipata* is necessary to generate the basis for efficient monitoring and management of this species. Determining the duration of the larval stage is particularly challenging because they feed inside the figs, making it difficult to follow their development. 

The first objective of this study was to determine several bioecological aspects of *S. adipata*. For this objective, a survey was taken in eight different sites in the states of Michoacán and Morelos, Mexico. The bioecological aspects investigated included the percentage and size of infested figs, number of larvae, pupae, and adults per fig, larva and pupa survival, and sex ratio. The second objective was to estimate and determine the duration of larval and pupal stages, respectively, of individuals from figs collected in the site with the highest percentage of infestation. The survival and longevity of adults were also determined.

## 2. Materials and Methods

Experiments were conducted in a controlled environment chamber at 25 ± 2 °C, 60 ± 5% relative humidity, and a photoperiod of 16:8 h (L:D). When conditions were different, we specifically detailed them below.

### 2.1. Insect Collection

From January to October 2023, unripe figs (cultivar Black Mission) were collected from fields in eight sites in two states of Mexico: seven sites in Michoacán and one in Morelos (Table 1). These collection sites were selected because they are the main fig production areas in these Mexican states. In each collection, a zig-zag sampling was performed between fig rows. Following collection, figs were placed in plastic boxes and transported to the Instituto de Investigaciones Agropecuarias y Forestales (IIAF), Universidad Michoacana de San Nicolás de Hidalgo (UMSNH), in El Trébol, Tarímbaro, Michoacán.

### 2.2. Percentage and Size of Infested Figs; Number of Larvae, Pupae, and Adults per Fig; Larva and Pupa Survival, and Sex Ratio

In the laboratory, figs from each collection site were individually placed into ventilated plastic cups (125 mL; Orox-co^®^, San Pedro Tlaquepaque, Jalisco, Mexico) containing vermiculite as a pupation substrate for *S. adipata* larvae. These cups were examined daily; then, the number of larvae that left the figs to pupate in the vermiculite was recorded. The percentage of infested figs was calculated by dividing the number of infested figs by the total number of collected figs × 100. One fig was considered infested when at least one larva came out of it to pupate.

To determine the size of infested figs, the length and width of collected figs were measured using a Vernier (Truper^®^, Model Caldi-6MP; Jilotepec, Estado de México, Mexico) before placement in the ventilated plastic cups described above. For length, each fig was measured from the peduncle to the base of the ostiole (distal end), and, for width, each fig was measured at its widest part perpendicular to its peduncular axis. The number of larvae per fig and the total number of larvae per collection site were also assessed. After larvae emerged from the figs to pupate in the vermiculite, each fig was dissected and examined under a stereoscopic microscope, looking for larvae, pupae, or adults inside them.

After 6–8 h of pupation, pupae were individually placed in cylindrical wells of 24-well Castor tissue culture plates (Corning^®^, New York, NY, USA) containing vermiculite and checked daily until adult emergence. The survival of larvae and pupae, for each collection site, was determined by dividing the number of larvae or pupae that molted to the next development stage by the initial number of larvae or pupae × 100 [23]. To this, larvae and pupae were assessed daily. After adult emergence, the sex ratio was calculated as the percentage of females in the population $[females/\left(females+males\right)\times 100]$. *Silba adipata* adults were identified using the keys of McAlpine [24].

### 2.3. Duration of Larval and Pupal Stages and Adult Survival and Longevity

To evaluate these biological parameters, individuals obtained from figs collected in the Los Tejones site were used. The number of recovered larvae in this site was the highest (*N* = 103) of all the sites (as detailed in the Results Section). Duration of the larval stage was estimated as the difference between the day of pupation and the day on which the infested figs were collected in the field. To determine duration of the pupal stage, they were checked daily.

After emergence, adults were released into a frame box (25 cm× 25 cm× 25 cm) entirely covered by a mesh screen and fed a semisynthetic diet based on sugar and hydrolyzed protein (3:1). The diet was placed in a Petri dish (9 cm diameter × 1.5 cm height) and replaced every seven days. Purified water was also offered continuously to adults with a piece of cotton in a Petri dish (5 cm in diameter by 1.5 cm in height). When adults were 15 days old, four ripe figs were offered as oviposition substrates and replaced every two days for one month. To determine survival and longevity, *S. adipata* adults (distinguishing between males and females) were assessed every 24 h until they died. Development of adults was followed under laboratory conditions of ~25 °C, 56% relative humidity, and a photoperiod of 12:12 h (L:D), with daylight entering from the room windows, as in Katsoyannos [18]. In addition, previous tests had shown that, under the controlled environment conditions mentioned above, *S. adipata* adults died on the second or third day after emergence.

### 2.4. Data Analysis

A generalized linear model procedure (PROC GLM), with the LSMEANS test (*p* < 0.05) to separate means, was used for all analyses, except for evaluation of adult survival and longevity, as detailed below. A binomial distribution model was used to evaluate the percentage of infested figs, larva and pupa survival, and sex ratio of adults. Except for percentage of infested figs, data on size of infested figs, number of larvae per fig, larva and pupa survival, and sex ratio of adults were analyzed in figs from the collection sites where infestation of figs was >6% (Telixtac, El Carrizal, Plan de Ayala, and Los Tejones), as detailed in the Results Section. All statistical tests were performed using SAS/STAT (version 9.4; SAS Institute, Cary, NC, USA), and all data are expressed as mean ± standard error (SE). All the analyses were performed without transforming data because they met the assumptions of normality (PROC UNIVARIATE) and homoscedasticity (PROC GLM).

We used Gehan–Breslow Kaplan–Meier (K-M) survival analysis and the non-parametric procedure LIFETEST to compare survival curves between sexes of *S. adipata* adults. A pairwise multi-comparison procedure (Long-Rank test, *p* < 0.05) was used to detect significant differences. We considered all female and male survivors from the beginning to the end of the experiment. Data on adult longevity were subjected to Student’s *t*-test.

## 3. Results

### 3.1. Percentage and Size of Infested Figs; Number of Larvae, Pupae, and Adults per Fig; Larva and Pupa Survival, and Sex Ratio

#### 3.1.1. Percentage of Infested Figs

The percentage of figs infested by *S. adipata* was significantly higher in the Morelos site (33.3%; Telixtac) than in the six Michoacán sites (F_6,866_ = 11.6; *p* < 0.0001; Table 2), with the exception of El Carrizal (24.5%; *p* = 0.1551). The percentage of infested figs was very similar in Plan de Ayala and Los Tejones (17.5–18.4%), whereas in Antúnez, Tangancícuaro, and Indaparapeo, it was very low (≤5.8%). No infestation was recorded in Charapendo.

#### 3.1.2. Size of Infested Figs

Significant differences were observed in the length of the infested figs collected in the four collection sites analyzed (F_3,97_ = 10.51; *p* < 0.0001; Table 2). The figs collected in El Carrizal and Plan de Ayala were significantly longer (3.4 and 3.5 cm, respectively) than those collected in Telixtac and Los Tejones (2.9 and 3.0 cm, respectively). Significant differences were also observed in the width of the infested figs from the different collection sites (F_3,97_ = 5.95, *p* = 0.0009; Table 2). Figs collected in Los Tejones and Telixtac were significantly wider (2.2 and 2.3 cm, respectively) than those collected in El Carrizal and Plan de Ayala (1.9 and 2.0 cm, respectively).

#### 3.1.3. Number of *S. adipata* Larvae, Pupae, and Adults per Fig

Figs collected in Plan de Ayala and El Carrizal had significantly (F_3,97_ = 6.13; *p* = 0.0007) more larvae (2.9 and 3.5 per fig, respectively) than those collected in Telixtac and Los Tejones (1.6 and 2.1 per fig, respectively; Table 2).

After the figs were dissected, only pupae or only adults of *S. adipata* were found in some, but both development stages of this insect were found in twelve of the figs collected in Telixtac (two and nine figs had two and one pupa each, respectively, whereas one fig had an adult). In addition, three of the collected figs in Los Tejones had one pupa each.

#### 3.1.4. Larval and Pupal Survival and Sex Ratio

The survival of *S. adipata* larvae (between 90.5% and 97.2%) and pupae (between 85.7% and 94.3%) obtained from the figs collected in El Carrizal, Plan de Ayala, Los Tejones, and Telixtac was very high (Table 3). No significant differences in survival were observed among individuals of each development stage recovered from figs collected in these collection sites (F_3,233_ = 0.58; *p* = 0.628 for larvae and F_3,217_ = 0.62; *p* = 0.601 for pupae).

No significant differences were detected in the adult sex ratio of the individuals obtained from El Carrizal, Plan de Ayala, Los Tejones, and Telixtac (F_3,187_ = 0.56; *p* = 0.6391). The proportion of females was between 40.0% and 53.5% (Table 3).

### 3.2. Duration of Larval and Pupal Stages and Survival and Longevity of Adults

#### 3.2.1. Larval and Pupal Duration

We recovered 103 *S. adipata* larvae from figs collected in Los Tejones. In this regard, 57 larvae left the figs to pupate in the vermiculite at 5 day or less after the figs were placed on this substrate (4, 12, 13, and 28 larvae on days 2, 3, 4, and 5, respectively), while 32 larvae left the figs to pupate between 6 and 11 d (12, 5, 4, 4, 5, and 2 larvae on days 6, 7, 8, 9, 10, and 11, respectively). Only eight larvae had the most extended duration (six came out of the figs at 13 d after collection and the other two larvae at 15 d after collection). If these eight larvae were the smallest ones at the moment of collection, they represent those of the earliest larval stage. Therefore, the duration of the *S. adipata* larval stage could be between 13 and 15 d. The pupal stage lasted 10.90 ± 0.06 d.

#### 3.2.2. Adult Survival and Longevity

**Figure 1:**
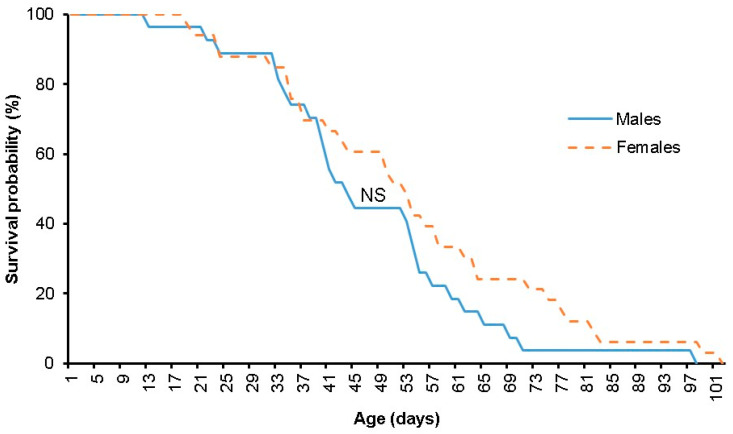
Survival probability of *Silba adipata* adults derived from figs collected in Los Tejones, municipality of Los Reyes de Salgado, Michoacán, Mexico. *NS*, no significant differences between sexes (*p* < 0.05). (*N* = 33 females and 27 males from the beginning of the experiment).

No significant differences in survival were observed between *S. adipata* males and females derived from figs collected in Los Tejones (Long-rank test, χ^2^ = 1.4657, *p* = 0.6902). The estimation of the survival rates of adults of this insect indicated that the probability of survival of an individual to 12 days was 100% in both sexes (Figure 1). Similar survival rates in males (96–70%) and females were observed from day 13 to day 39 (100–69%). Afterward, survival decreased with variation depending on the sex of the insect. At 50 days, the survival rate of females was 10% higher than that of males (54.5% vs. 44.4%). A 10% survival threshold was reached for males and females at 65 and 82 days, respectively. One male survived 97 days, while a single female survived 101 days.

No significant differences (*t* = −1.21, *p* = 0.231) in the longevity of adult *S. adipata* males and females were observed. The female (*N* = 33) and male (*N* = 27) adults lived for 52.6 ± 3.7 and 46.3 ± 3.4 days, respectively.

## 4. Discussion

Information on *S. adipata* fig infestation is very limited. Flores-Hernández [14] reported much higher infestation percentages (between 38 and 90%) in figs (cultivar Black Mission) collected in six sites in the Mexican states of Morelos and México than those found in our study (between 2 and 33%). In Tunisia, infestation by *S. adipata* on several fig cultivars ranged from 2 to 56%, 12 to 88%, and 0.01 to 81% in the regions of Djebba, Chott-Mariem, and Medenine, respectively [6]. These authors suggest that differences in fig infestation among several cultivars could be due to the pest preference/host susceptibility phenomenon, which may be related to fig color, size, skin texture, ostiole width, biochemical composition, and water content. In Mexico, the only other fig cultivar of economic importance is Brown Turkey; however, infestation of this insect on this cultivar is unknown. Therefore, it is important to conduct studies to determine whether this cultivar is more or less susceptible than the Black Mission cultivar.

Katsoyannos [18] mentioned that *S. adipata* preferably infests small and hard unripe figs; however, the size of the attacked figs was not given by this author. In the present study, we found that longer (~3.5 cm) and wider (~2.3 cm) unripe figs were more likely to be infested by this insect. This coincides with the size (3.2–3.8 cm long × 2.6–3.0 cm wide) of the unripe figs most infested [14] by *S. adipata* or by the related species *Silba virescens* Macquart (1–2.5 cm wide) [25]. Although *S. adipata* prefers to infest unripe figs [6,8,18], ripening figs can also be infested [18]. Our research group observed this in the laboratory, where females of this insect laid their eggs on ripening figs that were offered as oviposition substrates. Surprisingly, in the field, we also observed that *S. adipata* females laid eggs in the exit holes that larvae had drilled in the unripe figs. Even though we do not have a clear explanation for this oviposition behavior, we hypothesize that females of this insect use these exit holes to lay their eggs directly on the receptacle to ensure food for the larvae after their emergence. However, this merits further studies to understand this phenomenon fully.

The presence of the largest number of larvae per fig coincides with the biggest figs collected in the Plan de Ayala and El Carrizal sites. It is possible that a larger volume of tissue can provide enough food for a larger number of individuals. For instance, a higher number of lonchaeids *Dasiops inedulis* Steysdal and *Dasiops* spp. larvae were found in larger passion fruit (*Passiflora edulis* f. *flavicarpa* Degener) [26] and sweet granadilla (*Passiflora ligularis* Juss) [27] ovaries than in smaller ones.

It has been reported that when *S. adipata* larvae have reached their maximum development, they make a circular exit hole in the fig at the end of the mine where they have fed; they then fall out to pupate in the soil [6,10]. However, larvae that do not leave the figs can pupate inside and continue their development to adulthood. In our study, 18.2% and 1.1% of the collected figs in Telixtac and Los Tejones, respectively, contained pupae or adults. In addition, adults found inside the figs were dead and deformed (i.e., they were unable to shed the pupal exuvium or did not have normal wings). Similarly, Drouet [8] and Silvestri [28] reported that less than 1% of 1000 and 5000 dissected figs had pupae inside. The reasons that *S. adipata* pupate inside the figs are unknown, but we assume that these pupae are those formed from larvae with delayed development. In addition, the figs can become dehydrated, which prevents the larvae from leaving them to pupate in the soil.

We recorded a high survival rate of *S. adipata* larvae and pupae (≥86% in both development stages) of the individuals obtained from figs collected in the El Carrizal, Plan de Ayala, Los Tejones, and Telixtac sites. This insect is a monophagous species; therefore, the survival rate of these development stages could mean high adaptation to fig because it is its sole host. From a practical point of view, this finding could have negative implications because damage to fig crops caused by feeding larvae would increase. In addition, it is plausible to assume that if pupae have a high survival rate, then there will also be a high rate of adult emergence and, consequently, an increase in the offspring in the next generation. The survival of larvae and pupae in our studied species was higher than that reported for the lonchaeid *D. inedulis* (84% and 32% for larvae and pupae, respectively) [29]. The differences between the percentages of larvae and pupae survival obtained in our study and those obtained by Carrero [29] in *D. inedulis* could be intraspecific.

Our results regarding the sex ratio (~50% of the proportion of females) are practically the same as those reported in other studies with lonchaeid species: 50% in *D. inedulis* [26] and *Dasiops* spp. [27] were fed passion fruit and sweet granadilla ovaries, respectively. In contrast, a proportion of 60% of females was found in *Dasiops saltans* (Townsend) when fed yellow pitaya (*Selenicerus megalanthus* [Haw]) flower buds [30].

The biology of *S. adipata* is poorly understood. For the specific case of the larval stage, only two studies report the duration of this development stage [8,28], but no information is provided on how this biological parameter was determined. One of the studies reported that in spring, the larva of this species lasted 24 d, and in summer, it may last 6 to 7 d [28], while in the other study, the larva lasted 22 to 29 d in spring and 7 to 22 d in summer [8]. The estimated duration of the *S. adipata* larval stage obtained in our study was between 13 and 15 d. However, it is important to point out that this estimation does not represent the total duration of this development stage because the time at which we began following development was highly variable (between 2 and 15 d) among the individuals since different larval instars were found inside the figs at the moment they were collected.

In other lonchaeid species, the duration of the larval stage depends on both temperature and food. For example, in *S. virescens*, it was 32, 18, 10, and 7 d at 15, 20, 25, and 30 °C, respectively, when fed green figs [25]. In *Lonchaea chorea* (Fabricius) the stage lasted 60–72 and 12 d at 4.5–10 and 21–25 °C, respectively, when fed fresh cow dung [31], while in *Neosilba perezi* (Romero & Ruppel), it lasted 15.8 d at 22 ± 1 °C when fed an artificial diet of cassava flour, water, and brewer’s yeast [32]. However, in *N. perezi*, there was high larval mortality (46–52%) when they were fed two different artificial diets (one of them consisting essentially of brewer’s yeast + water + casein + agar and the other based on cassava flour + water) [32], suggesting that the food supplied was of low quality. It is well known that environmental conditions, especially temperature [8] and the type of diet [32], can strongly influence the development of insects. High temperatures within the optimal developmental range may accelerate metabolism and shorten development time. Moreover, the nutritional value of proteins, carbohydrates, and other dietary components influences the efficacy of their utilization and affects insect development [33]. In addition, the influence of these factors is species-dependent.

There are only two studies on the duration of the *S. adipata* pupal stage [8,28]. Based only on observations, these authors reported that the duration of the development stage of this insect was very similar in both spring and summer (9–10 d), but in the autumn, it was 16 d [8]. In other species of loncheids, this biological parameter was dependent on the environmental conditions: in *S. virescens*, it was 33, 21, 10, and 9 d at 15, 20, 25, and 30 °C, respectively [25]; in *L. chorea*, it was 10 d at 21–25 °C [31]; and in *N. perezi*, it was 23 d at 22 ± 1 °C [32]. The results on the duration of the pupal stage in *S. virescens* [25] and *L. chorea* [31] (10 d in both cases) at 25 °C and 21–25 °C, respectively, are very similar to those obtained in our study (11 d) at the same temperature.

To the best of our knowledge, there are no references to the survival of adults of *S. adipata*. In our study, the survival probability of females of this species under our conditions (~25 °C) dropped below 50% at 53 d old. In other tephritoids, this survival percentage was reached when the females were younger than our studied species: 19 d in *Anastrepha ludens* (Loew), 24 d in *Anastrepha obliqua* (Macquart), and 27 d in *Anastrepha serpentina* (Wiedemann) [34]. In contrast, a 50% survival threshold was obtained in *Anastrepha distincta* (Greene) females when they were 140 d old [35]. It has been reported that extended survival clearly influences the reproductive parameters in insects [34,36,37]. For example, in the tephrids *Anastrepha fraterculus* (Wiedemann), *Ceratitis capiatata* (Wied), and *Bactrocera dorsalis* (Hendel), the number of eggs laid by a female during her lifetime was 336, 706, and 1244, respectively [38,39]. In addition, a high fertility rate (88%) was recorded in *A. fraterculus* and *C. capitata* [38]. The fecundity and fertility of *S. adipata* have not been studied thus far. In preliminary tests, we recovered 30 eggs from one of the four ripening figs that were offered as oviposition substrates to the females of this insect. These eggs, however, could have come from more than one female, and they did not hatch. It may be that females of this insect were unmated, as suggested by Katsoyannos [18], who recovered 2000 non-viable *S. adipata* eggs from unripe figs. Further research should be implemented to understand this aspect completely.

In our study, no differences were observed in the longevity between sexes in *S. adipata*. However, females lived, on average, seven days more than males when provided with a semisynthetic diet based on sugar and hydrolyzed protein + water. Similarly, no significant differences were observed between *C. capitata* females and males (32 vs. 24 d) when they were fed a diet based on autolyzed yeast and sugar (1:99) [40]. Sugar and hydrolyzed protein provide different nutrients, as the former are carbohydrate sources, and the latter are protein sources. This could have contributed to the prolonged longevity of adults of both sexes of *S. adipata* in our study. In the field, adults of this species can obtain significant amounts of carbohydrates from the exudates of ripe figs [18]; this has been confirmed by our observations in the field and under laboratory conditions where ripe figs were offered for oviposition. However, if ripe figs are not available, *S. adipata* adults can obtain carbohydrates from other sources such as milky fig tree sap or flowers of the trumpet creeper plant (*Campsis* [*Tecoma*]) *radicans* Juss.; Bingoniacae) and citrus and olive trees [18].

## 5. Conclusions

Our study has shown several important ecological features of *S. adipata* derived from individuals collected from the field in eight sites of the Mexican states of Michoacán and Morelos. One of the main findings was that, in general, infestation by this insect was higher in the figs collected in the Morelos site than those collected in the Michoacán sites and that longer and wider figs were the most infested and had more larvae per fig. Moreover, this is the first study where the larval duration time was estimated and the duration of the pupal stage and the survival and longevity of *S. adipata* were determined. *Silba adipata* presents a serious challenge to the ongoing development of integrated pest management in fig crops. Therefore, currently, we are conducting more studies to develop a semisynthetic diet to determine the duration of the development stages of the egg and larva of this insect and its reproductive parameters regarding fecundity and fertility.

## Figures and Tables

**Table 1:** Location of collection of figs used in the study.

Collection Site	Municipality	Coordinates (Meters Above Sea Level)	Collection Date
Plan de Ayala	Los Reyes de Salgado	19°32′53.6″ N, 102°28′22.9″ W (1347)	4 January 2023
Charapendo	Gabriel Zamora	19°15′43.2″ N, 102°03′27.6″ W (974)	14 January 2023
Indaparapeo	Indaparapeo	19°47′35.6″ N, 100°58′46.2″ W (1889)	17 January 2023
Antúnez	Parácuaro	18°59′36.2″ N, 102°12′18.1″ W (340)	6 Febrary 2023
El Carrizal	Susupuato	19°13′35.7″ N, 100°26′13.5″ W (1177)	8 Febrary 2023
Tangancícuaro	Tangancícuaro	19°51′22.9″ N, 102°11′37.5″ W (1733)	20 March 2023
Los Tejones	Los Reyes de Salgado	19°32′01″ N, 102°34′41″ W (1053)	22 August 2023
Telixtac	Axochiapan	18°34′10″ N, 98°46′38″ W (1115)	2 October 2023

All collection sites are in Michoacán, except for Telixtac, which is in Morelos.

**Table 2:** Fig infestation percentage, size of infested figs, and number of larvae per fig in collection sites in the states of Michoacán and Morelos, Mexico.

Collection Site	Infestation (%; *N*)	Size of Infested Figs (cm)		Larvae/Fig
		Length	Width	
Telixtac	33.3 ± 5.8 (66) a	2.9 ± 0.07 b	2.3 ± 0.06 a	1.6 ± 0.30 b
El Carrizal	24.5 ± 6.2 (49) ab	3.4 ± 0.10 a	1.9 ± 0.08 b	3.5 ± 0.40 a
Plan de Ayala	18.4 ± 3.8 (103) b	3.5 ± 0.08 a	2.0 ± 0.06 b	2.9 ± 0.32 a
Los Tejones	17.5 ± 2.3 (275) b	3.0 ± 0.05 b	2.2 ± 0.04 a	2.1 ± 0.20 b
Antúnez	5.8 ± 2.0 (139) c	_	_	_
Tangancícuaro	5.0 ± 2.0 (119) c	_	_	_
Indaparapeo	1.6 ± 1.2 (122) c	_	_	_

Means followed by the same letter in the same column are not significantly different (LSMEANS test, *p* < 0.05). *N*, the total number of collected figs. _, data not included in the analysis because fig infestation was ≤6%.

**Table 3:** Survival of larvae and pupae and adult sex ratio of *S. adipata* obtained from infested figs collected in different sites in the states of Michoacán and Morelos, Mexico.

Collection Site	Larva (*N*)	Survival (%)		Sex Ratio (% Female)
		Larva	Pupa	
Telixtac	36	97.2 ± 4.2 a	85.7 ± 5.0 a	40.0 ± 9.1 a
El Carrizal	42	90.5 ± 3.9 a	89.5 ± 4.8 a	52.9 ± 8.7 a
Plan de Ayala	56	94.6 ± 3.4 a	94.3 ± 4.0 a	51.2 ± 7.9 a
Los Tejones	103	92.2 ± 2.5 a	90.5 ± 3.0 a	53.5 ± 5.4 a

Means followed by the same letter in the same column are not significantly different (LSMEANS test, *p* < 0.05). *N*, number of specimens whose development was followed.

## Data Availability

The data sets generated during and/or analyzed during the study are available from the corresponding author upon reasonable request.
